# Fullerene-Passivated Methylammonium Lead Iodide Perovskite Absorber for High-Performance Self-Powered Photodetectors with Ultrafast Response and Broadband Detectivity

**DOI:** 10.3390/molecules30051166

**Published:** 2025-03-05

**Authors:** Lakshmi Praba, Yoseob Chung, Dong Ho Han, Jae Woong Jung

**Affiliations:** 1 Department of Advanced Materials Engineering for Information and Electronics, Kyung Hee University, 1732 Deogyeong-daero, Giheung-gu, Yongin-si 446-701, Republic of Korea; plpraba97@khu.ac.kr (L.P.); cys1848@gmail.com (Y.C.); dhk916@khu.ac.kr (D.H.H.); 2 Integrated Education Institute for Frontier Science and Technology (BK21 Four), Kyung Hee University, 1732 Deogyeong-daero, Giheung-gu, Yongin-si 446-701, Republic of Korea

**Keywords:** perovskite, photodetectors, passivation, defect density, responsivity, detectivity

## Abstract

We herein report the enhanced electrical properties of self-powered perovskite-based photodetectors with high sensitivity and responsivity by applying the surface passivation strategy using C_60_ (fullerene) as a surface passivating agent. The perovskite (CH_3_NH_3_PbI_3_) thin film passivated with fullerene achieves a highly uniform and compact surface, showing reduced leakage current and higher photon-to-current conversion capability. As a result, the improved film quality of the perovskite layer allows excellent photon-detecting properties, including high values of external quantum efficiency (>95%), responsivity (>5 A W^−1^), and specific detectivity (*>*10^13^ Jones) at zero bias voltage, which surpasses those of the pristine perovskite-based device. Furthermore, the passivated device showed fast rise (0.18 μs) and decay times (17 μs), demonstrating high performance and ultrafast light-detecting capability of the self-powered perovskite-based photodetectors.

## 1. Introduction

Photodetectors (PDs) play a critical role in a wide range of optoelectronic devices, including optical signal receivers and optical communication systems for industrial applications [1,2,3]. Currently, the majority of commercial PDs are manufactured using inorganic semiconductors such as silicon (Si), indium gallium arsenide (InGaAs), and gallium nitride (GaN). These materials can effectively absorb light across broad spectral regions, ranging from the ultraviolet (250–400 nm) and visible (450–800 nm) to the infrared (900–1700 nm) [4]. Although inorganic semiconductor-based PDs have been widely adopted due to their reliable performance, their mechanical rigidity and complex fabrication processes limit their use in emerging smart electronics, such as wearable and deformable devices. Moreover, the performance of state-of-the-art inorganic PDs does not meet the requirements of modern applications, leading to intensified research efforts to discover novel photoactive semiconductors with fast photoresponse, high sensitivity, and high detectivity under low-light conditions [5].

Organolead trihalide perovskites have garnered significant attention as promising photoactive semiconductors due to their large optical absorption coefficient, long charge carrier lifetime, and diffusion length, as well as low material cost, which strongly illustrate their considerable potential in PD applications [6,7,8,9,10]. However, perovskite thin films produced through solution processing often exhibit polycrystalline surfaces with numerous grain boundaries, amorphous regions, and defect sites. Studies indicate that solution-processed perovskite films typically possess high trap-state densities (greater than 10^15^ per cubic centimeter) compared to ideal single-crystal counterparts (~10^9^ per cubic centimeter) [11]. These defects act as recombination centers, significantly reducing the films’ electronic performance [12,13,14]. Additionally, surface trap states in perovskite films promote non-radiative recombination of photo-generated carriers, leading to energy losses and diminished steady-state charge transport during continuous device operation. To enhance the film quality and performance of organolead trihalide perovskites, several strategies have been proposed, including composite engineering, optimized processing conditions, and the incorporation of additives during crystallization [15]. These approaches have been shown to reduce defect densities in perovskite thin films and improve the electronic properties of corresponding photovoltaic devices. While some processing techniques have been applied to improve the film quality and electronic performance of perovskite-based PDs, only a limited number of successful strategies have been demonstrated for achieving high-performance perovskite PDs [16].

Surface passivation is a widely explored approach to enhance the film quality of organolead trihalide perovskite thin films. The distorted lattice structure of [PbI_6_]^4−^ can create defective antisites in these perovskites, prompting the adoption of various passivating agents in perovskite photovoltaics [17]. Among the candidates, conjugated electron acceptors have shown potential as effective passivating molecules due to their ability to accept free electrons from undercoordinated dangling bonds or cation vacancies. For instance, fullerene derivatives have been utilized as Lewis acids to passivate undercoordinated iodide anions and PbI_3_^−^ antisites in perovskite films, thereby stabilizing the films and enhancing their photovoltaic performance [18]. Previous research by Huang and co-workers have demonstrated the effective passivation of perovskite absorber films using fullerene to mitigate trap sites in perovskite thin films. Xing et al. [19] also reported the use of novel fullerene derivatives for passivating perovskite thin films, effectively reducing surface trap states [20]. Sargent and co-workers further demonstrated that PC_61_BM could be selectively incorporated at the grain boundaries of polycrystalline perovskite films, effectively passivating halogen-rich trap sites [21]. Additionally, Huettner and co-workers suggested that PC_61_BM molecules could diffuse into the perovskite layer to passivate iodine-related defects [22]. These findings describe the critical role of fullerene-based passivation in reducing iodine-related vacancies and mitigating built-in field modulation, thereby enhancing charge transport efficiency in perovskite-based devices. However, a systematic investigation of this approach in photodetector (PD) applications to achieve high detectivity and responsivity remains unexplored.

Building on these insights, we propose a straightforward strategy to improve the electronic properties of perovskite photodetectors (PDs) by the fullerene passivation method. Dissolving fullerene in o-DCB enables simple solution-processed surface passivation of MAPbI_3_ layers, stabilizing defective antisites at the surface and grain boundaries to promote charge extraction and transport. The passivated films exhibited improved trap-state passivation, leading to PDs with a broad spectral response range (350 to 750 nm), high responsivity (5 A W^−1^), specific detectivity (2 × 10^13^ Jones), and fast response times (rise time of 0.18 μs and fall time of 17 μs) at zero bias. These self-powered perovskite PDs demonstrate enhanced electrical performance and light-detection capabilities across the UV–visible spectrum, offering a promising pathway for future device improvements.

## 2. Results and Discussion

**Figure 1:**
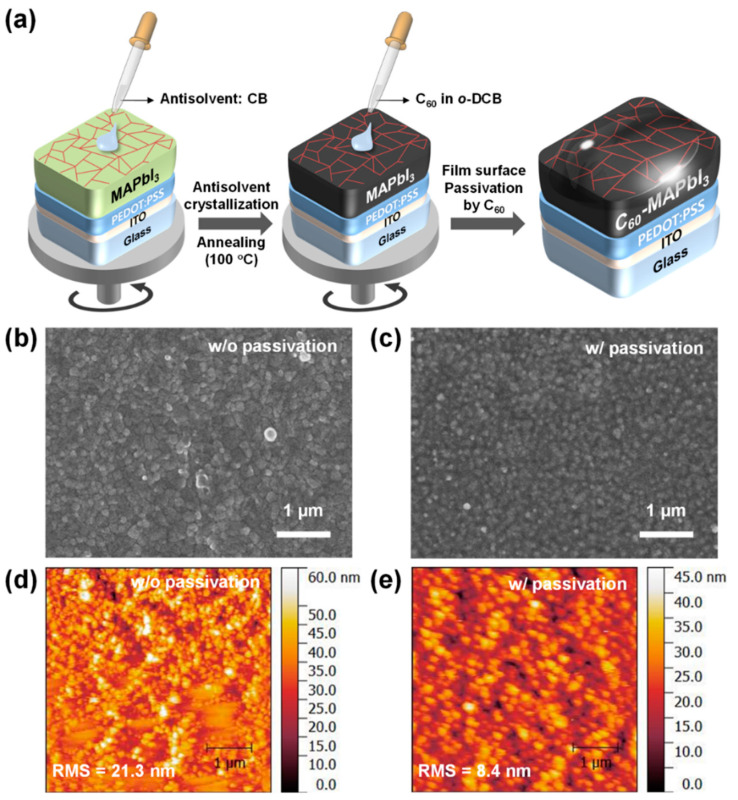
Fabrication process of the fullerene-passivated MAPbI_3_ films (**a**), SEM top-view images (**b**,**c**), and AFM topographic images (**d**,**e**) of MAPbI_3_ films without (**b**,**d**) and with (**c**,**e**) fullerene passivation.

As mentioned in the introduction, organolead trihalide perovskites demonstrate significant potential for photodetector (PD) applications due to their outstanding optoelectronic properties and compatibility with solution processing. In this study, polycrystalline MAPbI_3_ films were fabricated using the AAC method, followed by fullerene passivation via a dilute C_60_ solution in o-DCB (Figure 1a) [23]. The surface morphology of MAPbI_3_ perovskite films was analyzed with and without fullerene passivation. Figure 1b,c display FE-SEM images of both films, revealing a similar polycrystalline structure composed of compactly grown grains ranging from 200 to 250 nm in size (Figure S1). Additionally, atomic force microscopy (AFM) was used to further investigate the microstructural differences between the two films. As shown in Figure 1d,e, the AFM topography illustrates a significant reduction in root-mean-square (RMS) roughness, decreasing from 21.3 nm to 8.4 nm upon fullerene passivation. While the unpassivated MAPbI_3_ film exhibited some defective regions, the passivated film displayed a smoother and more uniform surface.

**Figure 2:**
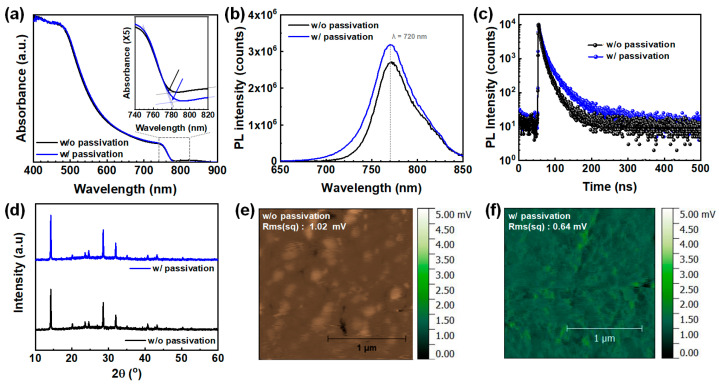
UV-Vis absorption spectra (**a**), photoluminescence spectra (**b**), PL decay graph (**c**), X-ray diffractograms (**d**), KPFM images for MAPbI_3_ with (**e**) and without fullerene passivation (**f**). Inset of (**a**) is a magnified absorption spectrum.

Due to its broadband absorption spanning from ultraviolet (UV) to the visible spectrum, MAPbI_3_ film serves as an effective photon-harvesting layer for UV-Vis broadband PDs (Figure 2a). The absorption spectra of both MAPbI_3_ films were largely similar, with only slight variations in their absorption onsets. However, Tauc plot analysis of the absorption spectra indicated that the optical band gap remained nearly identical for both films (Eg = 1.61 eV) (Figure S2). This confirms that fullerene passivation has a minimal impact on the optical absorption properties of MAPbI_3_ perovskite films. The negligible change in optical band gap between the two films was further supported by normalized steady-state photoluminescence (PL) spectra (Figure 2b), which showed PL peaks at 768 nm and 773 nm for the unpassivated and passivated films, respectively. The emission center shift of PL spectra toward a lower wavelength is attributed to the effective passivation of shallow trap sites present in the perovskite films. The increase in PL intensity upon passivation suggests that fullerene effectively suppresses non-radiative recombination at defect sites on the perovskite film surface [24]. To further examine the effect of passivation on carrier dynamics, time-correlated single-photon counting (TCSPC) measurements were conducted (Figure 2c). As summarized in Table S1, the PL lifetime increased from 35 to 68 ns with fullerene passivation, indicating a reduction in carrier recombination. The elongated PL lifetime and enhanced PL intensity demonstrate that fullerene passivation effectively modulates carrier recombination behavior, thereby influencing the electronic properties of the PD device. Additionally, X-ray diffraction (XRD) measurements were performed to assess the crystallinity of the perovskite films after passivation. As shown in the XRD diffractograms (Figure 2d), characteristic peaks were observed at 14.2°, 28.6°, 31.02°, and 43.38°, corresponding to the (110), (220), (310), and (330) crystal planes. The XRD analysis confirms that the perovskite films predominantly exhibit an orthorhombic crystal structure, regardless of fullerene passivation.

Kelvin probe force microscopy (KPFM) was further employed to examine the passivation effect of fullerene on the perovskite absorber. The local surface potential maps of the two films are presented in Figure 2e,f. KPFM enables precise measurement of local surface potentials by detecting contact potential differences (CPDs) between the probe tip and the sample surface, which are linked to their relative work functions (Figure S3). The results indicated negligible differences in CPD between the MAPbI_3_ films, regardless of fullerene passivation, suggesting that the electronic structure remained largely unaffected. However, variations in CPD across different grains were observed in the unpassivated MAPbI_3_ film, whereas the passivated film exhibited a more uniform surface potential. This suggests that fullerene passivation helps eliminate local potential wells, which could otherwise act as barriers to charge transport, thereby facilitating improved charge carrier mobility [25].

**Figure 3:**
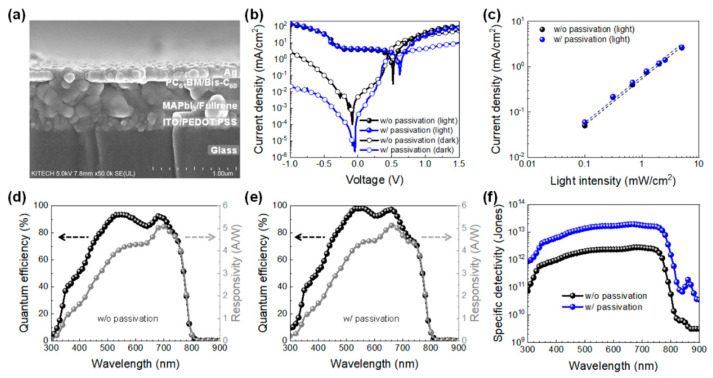
Cross-sectional SEM image (**a**), J–V curves (**b**), photocurrent density upon varied light intensity (**c**), EQE and responsivity spectra (**d**,**e**), and detectivity spectra (**f**) of the devices with and without fullerene passivation.

To evaluate the impact of fullerene passivation on the performance of perovskite PDs, we fabricated devices using a p-i-n structure. The cross-sectional SEM image, shown in Figure 3a, confirms the device architecture. The perovskite PDs adopt a sandwich-like p-i-n configuration, incorporating PEDOT:PSS as the hole transport layer and PC_61_BM as the electron transport layer. ITO and Ag were employed as the anode and cathode, respectively, to enhance the internal electric field, thereby facilitating exciton dissociation within the perovskite absorber layer. Additionally, Bis-C_60_ was introduced as a cathode modifier to suppress charge recombination and minimize leakage current under reverse bias—an essential factor for achieving high gain in photodetectors [26]. Figure 3b presents the current density–voltage (J–V) characteristics measured both in darkness and under illumination (100 mW cm^–2^, AM 1.5G), with a bias range from −1.0 V to 1.5 V. The pristine device exhibited a low dark current density in the range of 10^–3^ to 10^–4^ mA/cm^2^ at zero or low negative bias conditions (−0.1 V). This was further reduced to 10⁻^5^ to 10⁻^6^ mA/cm^2^ after fullerene passivation, primarily due to a decrease in defect density within the MAPbI_3_ layer. Further insights were gained through space-charge-limited current (SCLC) measurements of the electron-only devices, as detailed in the Supplementary Data (Figure S4). The trap-filled limit voltage (*V*_TFL_) decreased from 0.27 V to 0.20 V after passivation, leading to a corresponding reduction in trap density from 1.04 × 10^14^ cm^–3^ to 7.70 × 10^13^ cm^–3^. These findings indicate that fullerene passivation effectively suppresses trap-assisted recombination, thereby stabilizing the dark current in the MAPbI_3_-based device.

The photocurrents of both devices were comparable under varying white light illumination intensities (0.1 to 3 mW cm^–2^), as illustrated in Figure 3c. The relationship between photocurrent density and light intensity at 0 V follows a power-law dependence, expressed as *J*_ph_~*P^α^*, where *J*_ph_ represents the photocurrent density, *P* denotes the light intensity, and α is the exponent. The fitted curves yielded *α* values of approximately 1.06 and 1.02 for the devices without and with fullerene passivation, respectively. The slightly lower α value in the passivated device indicates a near-linear dependence of photocurrent on light intensity, suggesting a reduced recombination rate due to fullerene passivation in the MAPbI_3_ layer. This further confirms the beneficial effect of passivation in improving charge carrier dynamics within the device.

To evaluate the photodetecting performance of the device, two key parameters—spectral responsivity (*R_λ_*) and external quantum efficiency (*EQE*)—were primarily analyzed. To determine the optimal fullerene concentration for passivation, the *EQE* spectra of devices prepared with varying fullerene concentrations (0.1 to 2.0 wt%) were compared. The results indicated that a 0.5 wt% solution was the optimal condition for passivating MAPbI_3_ films (Figure S5). Spectral responsivity measures the efficiency with which a photodetector responds to incident photons and is defined by the following equation:

Rλ (AW−1)=JphI=EQE·λ1240

In this equation, *J_ph_* represents the photocurrent density, *I* denotes the intensity of incident photons, and *EQE* corresponds to the quantum efficiency of the device at a given wavelength (*λ*). Figure 3d illustrates the *EQE* spectra and spectral responsivity of the pristine device across different wavelengths at zero bias. The non-passivated MAPbI_3_-based device exhibited a broad photoresponse ranging from 300 to 800 nm, with a peak EQE of 93% and spectral responsivities between 3 and 5 A W⁻^1^ within the 450–750 nm wavelength range. For the fullerene-passivated device, the EQE slightly increased to 97%, while the spectral responsivity remained similar (3–5 A W⁻^1^), indicating that the high crystalline quality of MAPbI_3_ ensures strong photoresponse performance regardless of passivation (Figure 3e). Another crucial performance metric for photodetectors is specific detectivity (D*). To compare the detectivity of both devices, calculations were performed under the assumption that shot noise is the dominant noise source [6,27]. As shown in Figure 3f, the detectivity was determined using the following equation:

D*=R·A122·e·Jdark12(JonesorcmHz12W−1)

In this equation, *R* represents the spectral responsivity, *A* is the active device area, *e* denotes the elementary charge, and *J_dark_* corresponds to the dark current. The detectivity of the fullerene-passivated device reached as high as 2 × 10^13^ Jones within the 350–760 nm range, significantly surpassing that of the pristine device (~10^12^ Jones) at zero bias. Another crucial parameter for assessing the electronic performance of photodetectors is the noise equivalent power (NEP). The NEP is an important figure of merit used to quantify the sensitivity of photodetectors, because it stands for the power of the light signal that generates a signal-to-noise (S/N) ratio of unity at an output bandwidth of 1 Hz, characterizing the detection limit of the detector [28]. NEP can be estimated using the measured dark current noise (*i_n_*) and spectral responsivity (*R_λ_*) of the devices, as expressed by the following equation:

NEP=inRλ

It is important to note that the noise current in photodetectors primarily consists of shot noise and thermal noise. However, for self-powered devices operating at 0 V, shot noise can be considered negligible. As a result, thermal noise is the dominant noise source in this study [29]. The thermal noise can be expressed using the following equation:

$$i_{n,t}=\sqrt{\frac{4k_{B}TB}{R}}$$

In this equation, *k*_B_ represents the Boltzmann constant, *T* is the temperature, and *R* is the resistance of the device, which is extracted from the dark current–voltage characteristics at 0 V. The calculated noise currents were 0.315 and 0.143 pA Hz^−1/2^ for the pristine and passivated devices, respectively. The corresponding NEPs were 0.063 pW for the pristine device and 0.028 pW for the passivated one, according to the equations. The significantly lower NEP of the passivated MAPbI_3_-based photodetector suggests that fullerene passivation enhances the device’s ability to detect light at very low intensities. Additionally, it outperforms both commercial silicon photodiodes and other perovskite-based photodetectors reported in the literature in terms of noise current and NEP [30].

In addition to NEP, the linear dynamic range (LDR) is another key metric commonly used in photodetector applications, such as image sensors. The LDR value characterizes the range of light intensities over which the photodetector maintains a constant responsivity. This relationship can be expressed as follows:

LDR=20log⁡Jph*Jd

In this equation, J*_ph_ represents the photocurrent of the device measured at a light intensity of 1 mW cm⁻^2^, and *J_d_* denotes the dark current. As shown in Figure 3c, both MAPbI_3_-based photodetectors exhibited a similar linear photoresponse at light intensities ranging from 0.03 to 4 mW cm⁻^2^. However, the LDR values for the devices were 64 dB for the pristine device and 110 dB for the fullerene-passivated device. Notably, the LDR of the passivated device exceeded that of an InGaAs photodetector (66 dB) and was comparable to a silicon photodiode (120 dB) [31]. Furthermore, it outperformed the perovskite PDs with interface engineering (~100 dB) reported in other studies [30]. This reinforces the significant enhancement of the optoelectronic properties of perovskite PDs, particularly in terms of detectivity and NEP, achieved through fullerene passivation.

**Figure 4:**
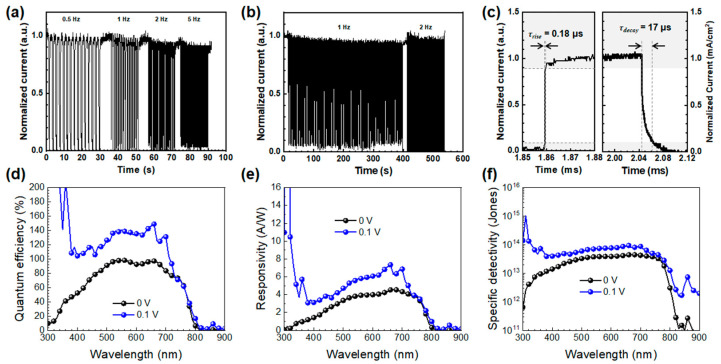
Temporal current response of the devices with varied light frequency (**a**), long-term measurement of current response (**b**), magnified photocurrent response at the on and off states (**c**), EQE (**d**), responsivity (**e**), and detectivity (**f**) of the fullerene-passivated device.

We then investigated the temporal response behavior of the fullerene-passivated perovskite PDs. Figure 4a shows the time-resolved photoresponse of the passivated PD under varying modulation frequencies of white light illumination (3 mW cm⁻^2^) at zero bias, demonstrating the device’s excellent photo-switching capability. The device exhibited a quick and reproducible photocurrent response with stable cycling performance, and as the modulation frequency increased from 0.5 to 5 Hz, the passivated device maintained a reliable photoresponse over a period of 600 s (Figure 4b). To assess the photoresponse speed, the time-resolved responses at the “on” and “off” states were magnified, as shown in Figure 4c. The sharp rise and fall edges of the response curve not only indicate a rapid response rate but also suggest efficient separation and collection of photo-generated carriers. The rise time (*τ_rise_*) is defined as the time taken for the photocurrent to increase from 10% to 90% of its steady-state value, while the decay time (*τ_decay_*) is the time it takes for the photocurrent to decrease from 90% to 10% of its steady-state value. In the passivated device, the rise and decay times were measured at 0.18 and 17 μs, respectively, reflecting the device’s ability to respond to fast optical signals in various applications. It is noteworthy that the response time of the fullerene-passivated PDs investigated in this study is considerably faster than that of other perovskite PDs reported elsewhere, as summarized in Supplementary Data (Table S2) [32,33,34,35,36,37,38,39]. The enhanced photoresponse performance of the fullerene-passivated MAPbI_3_ PDs can be attributed to a reduction in defect density at both the film surface and grain boundaries. This improvement facilitates efficient charge transport while concurrently minimizing charge recombination at the device interfaces.

Finally, we compared the photodetecting properties of the passivated device under different bias voltages to assess its capability for self-powered operation. The self-powered photodetectors should enable efficient photodetection without external power sources, making them highly attractive for sensor technologies. Figure 4d–f show a comparison of the EQE, responsivity, and detectivity of the passivated device at zero and –0.1 V bias. As expected, the device exhibited enhanced photon-to-electron conversion in the 300–700 nm range under the influence of a stronger external electric field. A similar trend was observed in the spectral responsivity. However, the magnitude of the enhancement was relatively modest, and the detectivity of the device at –0.1 V bias remained similar across the entire photon-harvesting range of MAPbI_3_ (~10^13^ Jones in the 350–750 nm range). Based on the EQE, responsivity, and detectivity data, it is evident that fullerene passivation is particularly beneficial for the device when operated without an external power source. This highlights the promising potential of fullerene passivation in further improving the performance of self-powered perovskite-based photodetectors, which is particularly advantageous for energy-efficient sensing applications such as environmental monitoring, biomedical diagnostics, and wearable technologies.

## 3. Experimental Procedures

### 3.1. Material

The chemicals and solvents used in this work were purchased from commercial sources and used as received. CH_3_NH_2_I (99%) was purchased from Greatcellsolar (Queanbeyan, Australia). PbI_2_ (perovskite grade) was purchased from TCI (Tokyo, Japan). Chlorobenzene (anhydrous, 99.8%), N,N-dimethylformamide (anhydrous, 99.8%), and dimethyl sulfoxide (anhydrous, 99.8%) were purchased from Sigma-Aldrich (St. Louis, MO, USA). Poly(3,4-ethylenedioxythiophene):polystyrene sulfonate (PEDOT:PSS) (Clevios P VP AI. 4083) was purchased from Baytron, H.C. Starck (Munich, Germany). Fullerene (C_60_) (99.9%) and Phenyl-C61-butyric acid methyl ester (PC_61_BM) (99.9%) were purchased from Nano-C (Westwood, MA, USA). Bis-C60 was synthesized as reported elsewhere [23].

### 3.2. Device Fabrication

ITO-coated glass substrates were cleaned with successive ultrasonication in deionized water, acetone, and isopropanol for 30 min, and then were dried by N_2_ gas blowing. After the ITO substrates were treated with UV-Ozone for 15 min, the PEDOT:PSS layer was deposited by spin-coating the aqueous solution at 4000 rpm for 60 s. The films were annealed at 150 °C for 30 min to evaporate the water. After the substrates were cooled down to room temperature, they were transferred to a N_2_-filled glove box. The perovskite precursor solution, which was freshly prepared by dissolving methylammonium iodide (224.2 mg) and lead(II) iodide (650 mg) in anhydrous dimethylformamid (0.897 mL), was mixed with 0.1 mL of anhydrous dimethyl sulfoxide. The perovskite layer was then deposited by spin-coating the precursor solution at 500 rpm for 5 s followed by 5000 rpm for 25 s. To obtain a highly crystalline but compact film, antisolvent-assisted crystallization (AAC) was adopted, where 0.1 mL of anhydrous chlorobenzene was dripped at the second step of spin-coating, followed by thermal annealing at 100 °C for 10 min. For the passivation of the perovskite surface, a solution of fullerene in *o*-dichlorobenzene (DCB) (0.5 wt%) was spin-coated on top of the perovskite film. The PC_61_BM solution in CB (15 mg/mL) and bis-C_60_ solution in isopropyl alcohol (2 mg/mL) were subsequently deposited as an ETL and a cathode modification layer, respectively. Finally, Ag (100 nm) was deposited by a thermal evaporator under the vacuum system 10^−6^ Torr to define the active area of the device (10 mm^2^).

### 3.3. Characterizations

The film surface morphologies were measured by field-emission scanning electron microscopy (FE-SEM) (MERLIN, Carl Zeiss, Oberkochen, Germany) and atomic force microscopy (AFM) (CoreAFM, Nanosurf) (Liestal, Switzerland) at the Core Facility Center for Analysis of Optoelectronic Materials and Devices of Korea Basic Science Institute (KBSI). The crystallinity of the perovskite layer was measured using an X-ray diffraction (XRD) using MiniFlex 300 (Rigaku, Tokyo, Japan). The optical spectra the films were measured by a UV-Vis spectrophotometer (Cary100, Agilent, Santa Clara, CA, USA) and a fluorescence spectrometer (FS5, Edinburgh Instruments, Livingston, UK). The picosecond laser with a wavelength of 405 nm (EPL-405, Edinburgh Instrument, Livingston, UK) was used in transient photoluminescence measurements. The electrical properties of the devices were obtained by a sourcemeter (4200-SCS, Keithley, OH, USA). The external quantum efficiency (EQE) of the device was measured with a chopped monochromatic light activated by a lock-in amplifier system under short-circuit conditions.

## 4. Conclusions

In summary, we investigated the effects of surface passivation on perovskite thin films in self-powered MAPbI_3_-based perovskite PDs by employing fullerene as a passivating agent. The introduction of fullerene passivation led to the formation of smoother, more uniform, and compact MAPbI_3_ thin films, which significantly improved the film’s surface morphology. This enhancement in film quality contributed to superior electronic properties by effectively suppressing charge recombination and minimizing leakage current within the device. As a consequence of these improvements, the fullerene-passivated devices exhibited a notable reduction in dark current density, along with enhanced EQE across the spectral range of 350 to 750 nm. These enhancements translated into excellent photodetection performance, with the devices achieving high responsivity values exceeding 5 A W^−1^ and a detectivity greater than 10^13^ Jones under zero bias voltage. Additionally, the fullerene-passivated devices demonstrated rapid photoresponse characteristics, with rise and decay times measured at approximately 0.18 μs and 17 μs, respectively, under zero bias. The results demonstrated in this work open up an opportunity for improving the electrical properties of the perovskite PDs in highly sensitive light-detecting applications that consume low amounts of energy, such as optical sensors, waveguide-integrated photodiodes, biosensors, or wearable optoelectronics.

## Data Availability

Data are contained within the article and Supplementary Materials.

## Supplementary Materials

The following supporting information can be downloaded at: https://www.mdpi.com/article/10.3390/molecules30051166/s1, Figure S1: Photographic images (a), SEM top-view images for MAPbI3 films without (b) and with fullerene-passivation (c); Figure S2: Tauc plots of MAPbI3 films with and without fullerene-passivation; Figure S3: Schematic illustrations of the KPFM measurement setup (a) and the electronic band structure at the grain boundary (b); Figure S4: SCLC graph of the devices with different MAPbI_3_ layers; Figure S5. EQE spectra of the devices with varied concentration of fullerene solutions on MAPbI_3_ films; Table S1. Summary of parameters from TRPL measurements of MAPbI_3_ layers; Table S2: Summary of device performance of MAPbI_3_-based photodetectors reported elsewhere.
