# Supplementary material for: Fullerene-Passivated Methylammonium Lead Iodide Perovskite Absorber for High-Performance Self-Powered Photodetectors with Ultrafast Response and Broadband Detectivity
Source: Molecules. 2025 Mar 5;30(5):1166. doi: 10.3390/molecules30051166 (PMC11901672; doi:10.3390/molecules30051166)
Supplement: Supplementary file 1 [file molecules-30-01166-s001.zip › molecules-3485714-supplementary.pdf]

Table S1. Summary of parameters from TRPL measurements of $MAPbI_3$ layers.

**passivation**	**t_1 (ns)**	**t_2 (ns)**	**t_{avg} (ns)**
w/o fullerene	15.47	68.83	35.35
fw/ fullerene	24.51	102.61	68.28

Table S2. Summary of device performance of $MAPbI_3$-based photodetectors reported elsewhere.

**Perovskite**	**R** (A W^{-1})	**D (Jones)**	**Rise/Decay Time**	**Operating voltage**	**Reference**
	5.1	2×10^{13}	-	0 V	This work
	13.57	5.25×10^{12}	-	−5 V	S1
	0.04	0.6×10^{12}	178 μs	5 V	S2
	4.95	2×10^{13}	0.1 ms	1 V	S3
$MAPbI_3$	1.2	2.39×10^{12}	10 ms	2 V	S4
	0.76	-	2.7 ms	0.4 V	S5
	1.2	3.07×10^{12}	2.21 μs	20 V	S6
	0.0022	1.76×10^{11}	27 ms	0V	S7
	0.52	8.8×10^{11}	19 μs	0V	S8

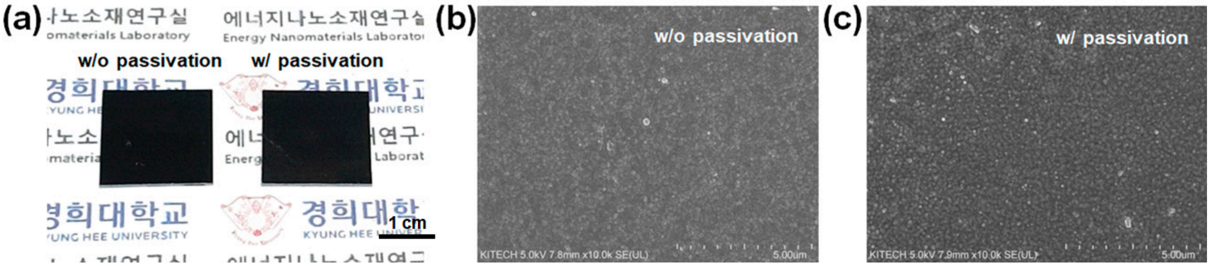

Figure S1. Photographic images (a), SEM top-view images for $MAPbI_3$ films without (b) and with fullerene-passivation (c).

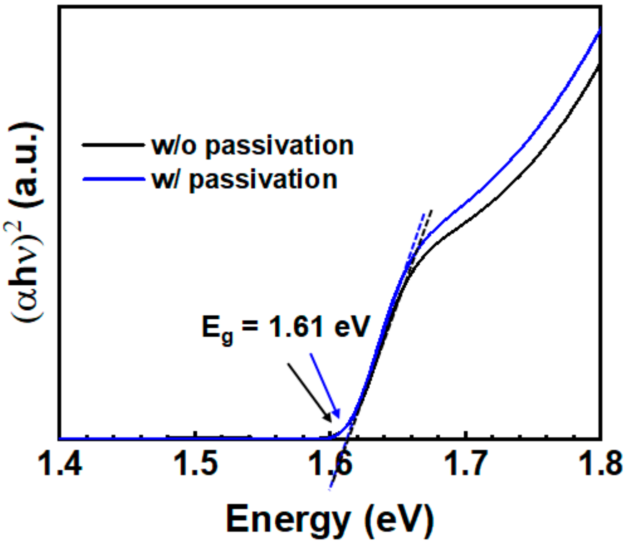

Figure S2. Tauc plots of $MAPbI_3$ films with and without fullerene-passivation.

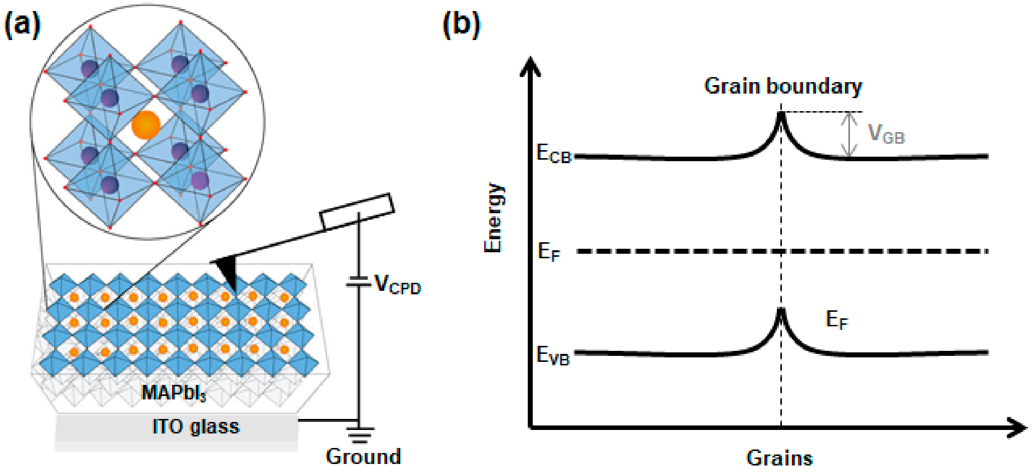

Figure S3. Schematic illustrations of the KPFM measurement setup (a) and the electronic band structure at the grain boundary (b).

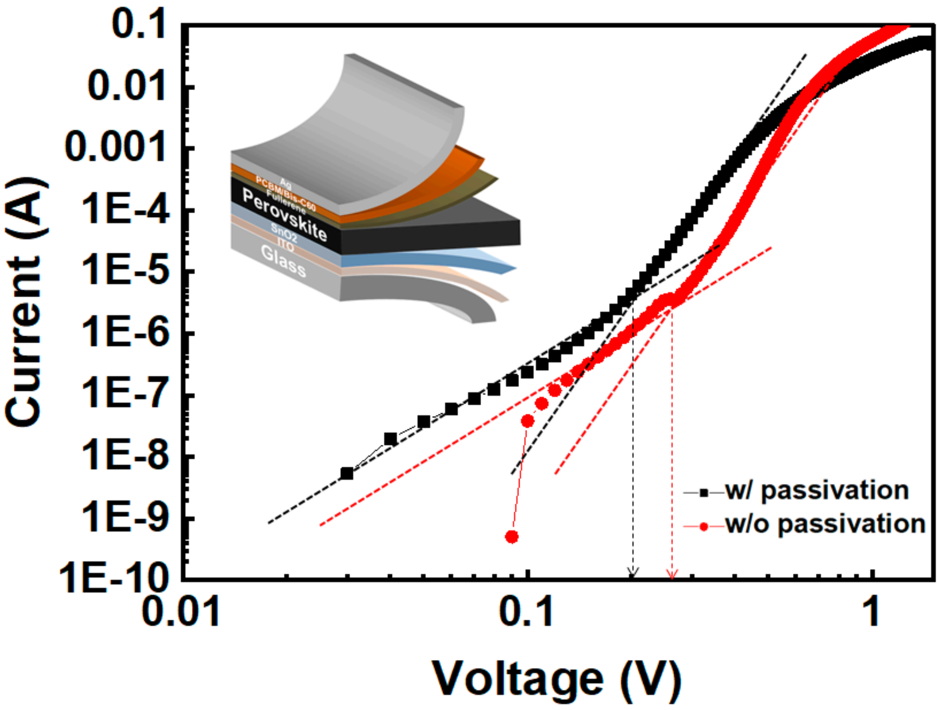

Figure S4. SCLC graph of the electron-only devices with different $MAPbI_3$ layers.

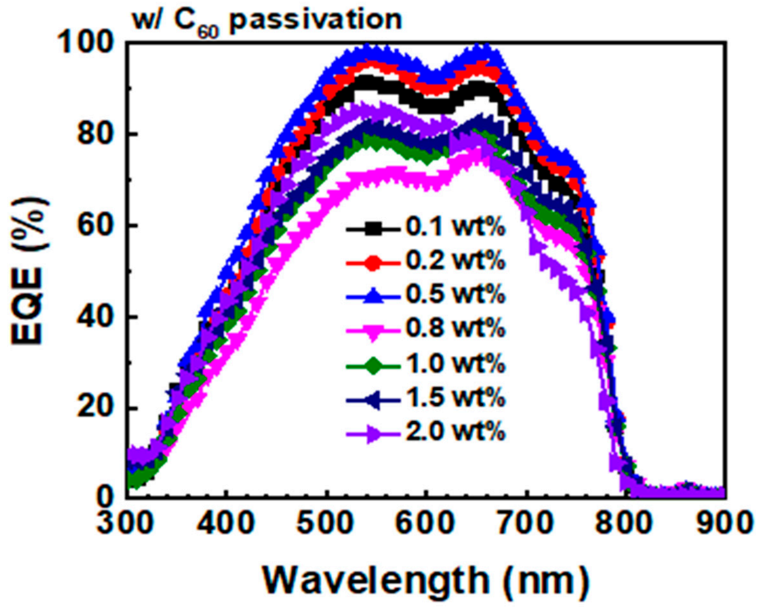

Figure S5. EQE spectra of the devices with varied concentration of fullerene solutions on $MAPbI_3$ films.

Supplementary references

[S1] W. Deng, X. Zhang, L. Huang, X. Xu, L. Wang, J. Wang, Q. Shang, S.-T. Lee, J. Jie, Aligned single-crystalline perovskite microwire arrays for high-performance flexible image sensors with long-term stability, Advanced Materials, 2016, 28, 2201–2208.

[S2] X. Yang, S. Hu, Y. Zhang, H. Li, C. Sheng, Methylammonium acetate as an additive o improve performance and eliminate J-V hysteresis in 2D homologous organic-inorganic perovskite solar cells, Solar Energy Materials and Solar Cells 2019, 191, 275–282.

[S3] L. Gao, K. Zeng, J. Guo, G. Ge, J. Du, Y. Zhao, C. Chen, H. Deng, Y. He, H. Song, G. Niu, J. Tang, Passivated single-crystalline $CH_3NH_3PbI_3$ nanowire photodetector with high detectivity and polarization sensitivity, Nano Letters 2016, 16, 7446–7454.

[S4] Y. Liu, F. Li, C. P. Veeramalalai, W. Chen, T. Guo, C. Wu, T. W. Kim, Inkjet-printed photodetector arrays based on hybrid perovskite $CH_3NH_3PbI_3$ microwires, ACS Applied Materials & Interfaces 2017, 9, 11662–11668.

[S5] R. Xiao, Y. Hou, Y. Fu, X. Peng, Q. Wang, E. Gnzalez S, Jin, D. Yu, Photocurrent mapping in single-crystal methylammonium lead iodide perovskite nanostructures, Nano Letters 2016, 16, 7710–7717.

[S6] Y. Xu, X. Wang, J. Zho, Y, Pan, Y. Li, E. E. Elemike, Q. I, X. Zhang, J. Chen, Z. Zhao, J. Akram, B. S. Bae, S. Bin, W. Lei, Solution-processed epitaxial growth of $MAPbI_3$, Frontiers in Materials 2021, 8, 651957.

[S7] S. Lim, M. Ha, Y. Lee, H. Ko, Large-area, solution-processed hierarchical MAPbI3 nanoribbon arrays for self-powered flexible photodetectors, Advanced Optical Materials 2018, 1800615.

[S8] G. R. Adams, V. O. Eze, M. A. S. Shohag, R. Simpson, H. Parker, O. I. Okoli, Fabrication of rapid response self-powered photodetector using solution-processed triple cation lead-halide perovskite, Engineering Research Express 2020, 2, 015043.
